# Prevalence of Depression, Suicidal Ideation, Alcohol Intake and Nicotine Consumption in Rural Central India. The Central India Eye and Medical Study

**DOI:** 10.1371/journal.pone.0113550

**Published:** 2014-11-19

**Authors:** Jost B. Jonas, Vinay Nangia, Marcella Rietschel, Torsten Paul, Prakash Behere, Songhomitra Panda-Jonas

**Affiliations:** 1 Suraj Eye Institute, Nagpur, Maharashtra, India; 2 Department of Ophthalmology, Medical Faculty Mannheim of the Ruprecht-Karls-University Heidelberg, Mannheim, Germany; 3 Department of Genetic Epidemiology in Psychiatry, Central Institute of Mental Health, Medical Faculty Mannheim of the Ruprecht-Karls-University Heidelberg, Mannheim, Germany; 4 Department of Psychiatry, Datta Meghe Institute of Medical Sciences, Wardha, Maharashtra, India; Mayo Clinic, United States of America

## Abstract

**Background:**

To investigate the prevalence of depression, suicidal ideations, alcohol and nicotine consumption in adults in an agrarian society mostly unchanged by the effects of urbanization.

**Methods:**

The Central India Eye and Medical Study is a population-based study in rural Central India close to the tribal belt and included 4711 subjects (aged 30+ years). Depression was assessed by the Center for Epidemiologic Studies Depression Scale (CESD), suicidal ideation by six standardized questions, nicotine use by the Fagerstroem Nicotine Tolerance Questionnaire (FTNQ), and alcohol consumption by the Alcohol Use Disorders Identification Test (AUDIT).

**Results:**

Mild to moderate depression (CESD sum score: 15–21) was detected in 1862 (39.6%) individuals (33.5% of men, 44.8 of women), and major depression (CESD sum score >21) in 613 (13.0%) individuals (8.1 of men, 17.3% of women). Suicide attempt was reported by 199 (4.2%) participants and suicidal thoughts during the last 6 months by 238 (5.1%) individuals. There were 887 (18.9%) smokers and smokeless tobacco was consumed by 1968 (41.8%) subjects. Alcohol consumption was reported by 1081 (23.0%) participants; 283 (6.0%) subjects had an AUDIT score ≥8 (hazardous drinking), and 108 (4.63%) subjects a score ≥13 (women) or ≥15 (men) (alcohol dependence).

**Conclusions:**

In rural Central India, prevalence of major depression was comparable to figures reported from other developing countries. Prevalence of smoking and hazardous alcohol consumption was higher than as reported from urban regions. Measures should be taken to address the relatively high prevalence of suicide attempts and thoughts on suicide in rural Central India.

## Introduction

Depression with a lifetime prevalence of 2% to 15% is one of the most important risk factors for substantial disability and is one of the major leading causes of disease burden in 2010 [1]–[5]. Smoking has become the leading cause of preventable death with yearly about 5 million premature deaths worldwide [1], [2], [6]–[8]. Alcohol consumption, among other risk factors such as dietary risks, tobacco smoking, high blood pressure, high fasting plasma glucose and physical inactivity, belongs to the leading risk factors related to DALYs [1], [2], [9]–[13]. Results from the Global Burden of Disease study showed that especially mental health disorders constitute an enormous and growing problem in developed and developing regions [14]. Despite the worldwide importance of depression, suicide, smoking and alcohol consumption, and although India is the second largest nation by the number of inhabitants, relatively little has been known about the prevalence of depression, suicide ideation, alcohol and nicotine consumption in India. It holds true in particular for the rural population in the under-developed regions of the Indian subcontinent. The present study was therefore conducted to assess the prevalence of depression, nicotine and alcohol abuse and possibly associated health issues in adults living in rural Central India in villages bordering the so-called tribal belt. Since the living conditions in these communities have not markedly changed within the last 100 years, the study additionally offered the possibility of assessing the prevalence of depression, smoking and alcohol consumption in an environment which has remained mostly untouched by factors associated with a developed technical and medical infrastructure.

## Methods

The Central India Eye and Medical Study (CIEMS) is a population-based cross-sectional study in Central India [15]. The Medical Ethics Committee of the Medical Faculty Mannheim of the Ruprecht-Karls-University Heidelberg and the ethical committee of Suraj Eye Institute/Nagpur approved the study and all participants gave informed written consent, according to the Declaration of Helsinki. The study was carried out in 8 villages in the rural region of Central Maharashtra at a distance of about 40 km from Nagpur. The villages were at the border of tribal regions. All villagers aged 30+ were invited to participate in the study which included a bus ride to the hospital, a one-day long examination, meals, and a return bus ride to the village in the evening all free of charge. All examinations were carried out on site in the hospital. Trained social workers interviewed the participants with the help of a standardized questionnaire including 200 questions on the socioeconomic background, living conditions, daily food, smoking or other types of tobacco consumption, alcohol consumption, amount and type of daily physical activity, known diagnosis of major diseases, and intake of medication. Addressing the questionnaire usually took about 30 to 40 minutes with the help of an experienced interviewer. If the study participants appeared to be fatigued, a break was made to make the study participant refresh.

The psychiatric status was assessed by the use of several questionnaires. Current depressive mood disorders were recorded by the Center for Epidemiologic Studies Depression Scale (CES-D) with a 20-item self-report depression scale [16]. A clinical episode of major depression was defined as a score of >21 in the CES-D. Six standardized questions concerning suicidal thoughts, suicidal behavior and suicidal attempts during the last 6 months and lifetime were asked. Individuals identified with suicidal thoughts were visited during the repeated visits to the villages as well as after the end of the study since the Suray Eye Institute continued serving visually handicapped villagers with cataract surgery. We used the Fagerstrom Test for Nicotine Dependence (FTND) to assess the smoking behavior [17]. In addition to the FTND, the history of former nicotine consumption (age of onset of tobacco consumption, cessation age, estimated pack years) and consumption of different tobacco products like hookah, bidis and smokeless tobacco products like snuff, chewing tobacco and bethel was recorded by 9 additional standardized questions. To determine alcohol consumption, the Alcohol Use Disorders Identification Test (AUDIT) was utilized [18]. The Audit is a 10-item self-report questionnaire with a sum score of 8 or more being associated with harmful or hazardous drinking, and a score of 13 or more in women and 15 or more in men, being likely to indicate alcohol dependence. In addition to the AUDIT, the history of former alcohol consumption (starting age of alcohol consumption, cessation age) and the current drinking behavior (e. g. type of drink) was assessed by a further 4 standardized questions.

Statistical analysis was performed using a commercially available statistical software package (SPSS for Windows, version 21.0, SPSS, Chicago, IL). We examined the mean values (presented as mean ± standard error for frequency parameters; presented as mean ± standard deviation for other parameters). All *P*-values were 2-sided and were considered statistically significant when the values were less than 0.05.

## Results

Out of total population of 13,606 villagers, 5885 subjects fulfilled the inclusion criterion of an age of 30+ years. Out of these 5885 subjects, 4711 people participated, resulting in a response rate of 80.1% (Table 1). The representativeness of the study population compared to the total population of India had been examined in previous investigations with no major difference detected between the population structure of the study and the general Indian population (Table 1) [15].

**Table 1:** Demographic data of the Central India Eye and Medical Study (Part I) and the whole of India according to the National Census - India (2001).

	Central India Eye and Medical Study			India (Total; in Millions)			India (Rural; in Millions)		
Age	Total	Males	Females	Total	Males	Females	Total	Males	Females
30–34 Years	405 (17%)	173 (16%)	232 (18%)	74.3 (19%)	37.4 (19%)	36.9 (19%)	51.8 (19%)	25.7 (18%)	26.1 (19%)
35–39 Years	381 (16%)	154 (14%)	227 (18%)	70.6 (18%)	36.0 (18%)	34.5 (18%)	49.0 (18%)	24.9 (18%)	24.1 (18%)
40–44 Years	336 (14%)	161 (15%)	175 (14%)	55.7 (14%)	29.9 (15%)	25.9 (14%)	38.6 (14%)	20.4 (15%)	18.1 (13%)
45–49 Years	256 (11%)	118 (11%)	138 (11%)	47.4 (12%)	24.9 (12%)	22.5 (12%)	33.0 (12%)	17.0 (12%)	15.9 (12%)
50–54 Years	248 (10%)	123 (11%)	125 (10%)	36.6 (9%)	19.9 (10%)	16.7 (9%)	25.8 (9%)	13.8 (10%)	12.0 (9%)
55–59 Years	141 (6%)	66 (6%)	75 (6%)	27.7 (7%)	13.6 (7%)	14.1 (7%)	20.0 (7%)	9.6 (7%)	10.3 (8%)
60–64 Years	214 (9%)	91 (8%)	123 (10%)	27.5 (7%)	13.6 (7%)	13.9 (7%)	20.7 (7%)	10.1 (7%)	10.5 (8%)
65–69 Years	172 (7%)	66 (6%)	106 (8%)	19.8 (5%)	9.5 (5%)	10.3 (5%)	14.8 (5%)	7.1 (5%)	7.7 (6%)
70–74 Years	176 (7%)	95 (9%)	81 (6%)	14.7 (4%)	7.5 (4%)	7.2 (4%)	11.1 (4%)	5.7 (4%)	5.4 (4%)
75–79 Years	48 (2%)	40 (4%)	8 (1%)	6.6 (2%)	3.3 (2%)	3.3 (2%)	4.8 (2%)	2.4 (2%)	2.4 (2%)
80+ Years	37 (2%)	27 (2%)	10 (1%)	8.0 (2%)	3.9 (2%)	4.1 (2%)	6.0 (2%)	3.0 (2%)	3.0 (2%)
Total	2414 (100%)	1114 (100%)	1300 (100%	388.9 (100%)	199.4 (100%)	189.5 (100%)	275.5 (100%)	139.8 (100%)	135.7 (100%)

There were 2520 (53.5%) women. Mean age of the study participants was 49.5±13.4 years (Table 2). Among the 1174 non-participants were 685 (58.3%) men; mean age was 48.6±14.1 years (median: 45 years; range: 30–95 years). The group of study participants and the group of non-participants did not differ significantly in age (*P* = 0.06), while the proportion of men was significantly (*P*<0.001) higher in the group of non-participants. Further details about the sub items of the non-psychiatric questionnaire, the socioeconomic background and the medical records were presented previously [15].

**Table 2:** Demographic Parameters and Data on Daily Activities, Smoking and Alcohol Consumption, and Depression in the Central India Eye and Medical Study.

Parameter	Mean ± Standard Deviation	Median	Range
Age (Years)	49.5±13.4	47.0	30, 100
Body Mass Index (kg/m^2^)	19.7±3.4	19.1	11.0, 46.3
Duration of Daily Work (Hours)	7.4±2.2	8	0.8, 18
Walking or Biking to Work (Days per Week)	6.6±1.2	7	1, 7
Diet	Purely vegetarians: 2394 (50.8%) subjects; Eating fish and eggs in addition to vegetables, but no meat: 35 (0.7%) subjects, Mixed diet: 2275 (48.3%) subjects		
Reported Monthly Income (Rupee)	1584±1233	1350	200, 15000
Family Income	<1500 Rupee: 2372 (50.5%); 1500–5000 Rupee: 2153 (45.8%); >5000 Rupee: 166 (3.5%)		
Family Type	Joint: 1968 (41.8%); Nuclear: 2607 (55.4%); Single: 133 (2.8%)		
Religion	Hindu: 4562 (96.9%); Muslim: 121 (2.6%); Others: 6 (0.6%)		
Level of Education	Illiterate: 1623 (34.5%); School up 5^th^ standard: 1310 (27.8%); School between 6^th^ to 8^th^ standard: 533 (11.3%); School between 9^th^ and 12^th^ standard: 1070 (22.7%); Higher level of education: 165 (3.5%)		
Possession of Mobile Telephone	139 (3.0%) participants		
Housing	Houses made out of mud (“kacha”): 1109 (23.5%); Houses made partially out of mud (“semi pucca”): 2330 (49.5%)		
House Ownership	Own house: 4486 (95.6%); Rented house: 194 (4.1%); Mortgaged: 13 (0.1%)		
Light Source	Electricity: 4315 (92.0%); Kerosene, gas or oil: 333 (3.5%);		
Room for Cooking in the House	Yes: 4209 (89.7%); No: 485 (10.3%)		
Toilet Facility	No toilet facility in the houses: 2206 (46.9%) subjects		
Water Source	Public tap, hand pump or well: 1719 (36.5%); Pipe, Hand pump or well in the residency/compound: 2904 (62.6%); Other: 18 (0.4%)		
Agricultural Land Ownership	No land; 2062 (44.0%); <2 acres: 482 (10.3%); 2.0–4.9 acres: 833 (17.8%); 5+ acres: 1313 (28.0%)		
Durable Goods Ownership	Mattress, pressure cooker, chair cot/bed, table or clock: 569 (14.3%); Bicycle, electric fan, radio/Transistor, sewing machine: 2941 (63.8%); Moped/scooter, motorcycle, fixed telephone or mobile telephone: 965 (20.9%); Car, tractor: 41 (0.9%); None: 92 (2.0%)		
Livestock Ownership	Yes: 1920 (41.0%); No: 2762 (59.0%)		
Irrigated Land Ownership	No: 2049 (58.8%); Owns at least some irrigated land: 1433 (41.2%);		
Depression Score CESD	14.1±7.5	15	0, 58
Smoking	Current and/or former smokers: 887 (18.9%) (883 men; 4 women); Former smokers: 184 (20.7%); Current smokers with unsuccessful try to quit: 398 (44.9%)		
Package Years	26.6±20.6	20.0	
Fagerstrom Test for Nicotine Dependence	3.87±2.5 points	4	
Fagerstrom Test for Nicotine Dependence	0–3: 424 smokers (49.6%); 4–6: 281 smokers (32.9%); 7–10: 150 smokers (17.5%)		
Smokeless Tobacco	1957 (mean daily consumptions: 4.23±2.2 times (median: 4)		
Alcohol Consumption			
No Alcohol	3628 (77.0%) participants		
consumption of alcohol once per month or less often,	287 (6.1%) participants		
alcohol consumption of 2–4 times/month,	264 (5.6%) participants		
alcohol consumption of 2–3 times/week	310 (6.6%) subjects.		
alcohol consumption of 4 times/week	166 (3.5%) subjects		
daily alcohol consumption	54 (1.1%) subjects		
AUDIT sum score (1081 individuals)	6.0±5.8 points (median: 4)		
AUDIT sum score of 8 or more (harmful or hazardous drinking)	283 subjects (6.0% of the study population/26.2% of the individuals consuming alcohol).		
AUDIT sum score of ≥13 in women or ≥15 or more in men (alcohol dependence)	108 subjects (4.6% of the study population; 51 women (8.9% of women consuming alcohol), 57 men (11.2% of men consuming alcohol).		

The mean body mass index was 19.7±3.4 kg/m^2^. Mean duration of daily work was 7.4±2.2 hours. The subjects went to work by foot or by bike in 6.6±1.2 days per week. 2394 (50.8%) subjects were purely vegetarians, 35 (0.7%) subjects reported to eat fish and eggs in addition to vegetables, but no meat, and 2275 (48.3%) subjects had a mixed diet. Mean reported monthly income was 1584±1233 Rupee (1 US$ was roughly the equivalent of 50 Rupee). The reported monthly family income was less than 1500 Rupee for 2377 subjects (50.4%). Hinduism was the predominant religion (97.2% of the study population). Out of the total 4711 subjects, 1623 (34.5%) subjects reported to be illiterate, 1310 (27.8%) subjects to have visited school up the 5^th^ standard, 533 (11.3%) subjects to have visited school between the 6^th^ to 8^th^ standard, 1070 (22.7%) subjects to have attended school between the 9^th^ and 12^th^ standard, and 165 (3.5%) subjects reported to have received a higher level of education such as graduation. Out of the 4711 subjects, 139 (3.0%) participants had a mobile. The majority of the study population lived in houses made out of mud without bricks and mortar (“kacha”; 1109 (23.5%) subjects) or made partially out of mud (“semi pucca2330 (49.5%) subjects). Out of the total 4711 subjects, 2206 (46.9%) subjects indicated that they did not have a toilet facility in their houses. Water source was a public tap, hand pump or well for 1719 (36.5%) subjects.

### Depression

Data on depression were available on 4698 (99.7%) subjects with a mean age of 49.5±13.4 years. The group of subjects with data on depression and the group of subjects without assessment of depression (mean age: 55.2±13.9 years) did not differ significantly in age (*P* = 0.67).

**Figure 1:**
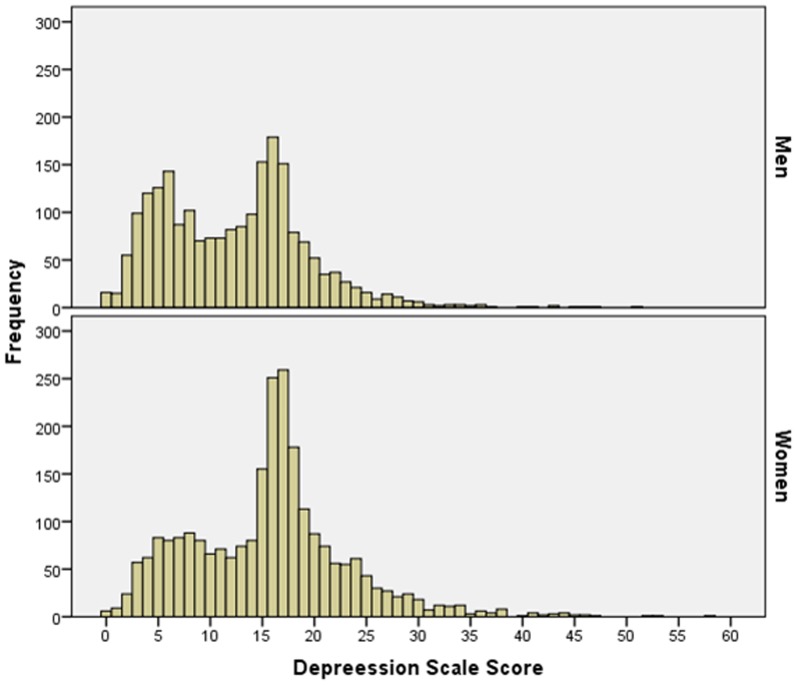
Histograms Showing the Distribution of the Depression Scale Score Stratified by Gender in the Central India Eye and Medical Study.

**Figure 2:**
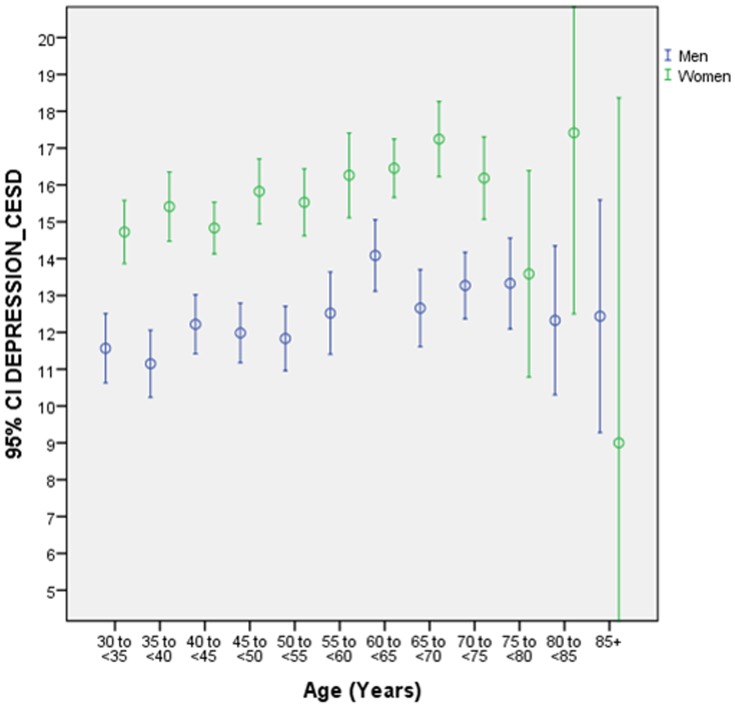
Graph Showing the Distribution of the Depression Score Stratified by Age and Gender in the Central India Eye and Medical Study.

The mean of the valid CESD sum scores was 14.1±7.5 points (median: 15; range: 0–58) (Fig. 1, 2). Men had a mean CESD sum score of 12.3±6.9 points (median: 13; range: 0–51), while female participants had a mean CESD sum score of 15.7±7.6 points (median: 16; range: 0–58). 1862 individuals (39.57%) showed a CESD sum score between 15 and 21 points indicating a mild to moderate depression within the last week, while 613 individuals (13.03%) scored >21 points in the CESD, indicating the possibility of a major depression. With respect to gender 734 men (33.52%) and 1128 (44.83) women scored between 15 and 21 points, while 178 men (8.13%) and 435 females (17.29%) scored more than 21 points in the CESD.

For 4702 (99.8%) of the 4711 participants three standardized questions concerning suicidal behavior and suicidal attempts during their lifetime could be assessed. 199 participants (4.2%) admitted to have made an attempt at suicide. Out of these, 79 (39.7%) were men. For 3890 of the 4711 participants, three standardized questions concerning suicidal thoughts during the last 6 months or earlier were recorded. Out of these, 238 (5.1% total; 83 men (34.9%)) reported to have thought of committing suicide in the last 6 months. The most often reported reason for attempts at suicide were family related (68.4%), financial problems (22.4%) and long illness (7.9%) for men, and family related (91.7%), financial problems (5.8%) and long illness (0.8%) for women. Reasons for thoughts of committing suicide were similar: family related (79.0%), financial problems (8.6%) and long illness (9.9%) for men, and family related (93.5%), financial problems (2.6%) and long illness (3.9%) for men.

### Smoking

With respect to smoking, 887 (18.84%) subjects reported to be or to have been smokers, with 184 (20.74%) being former smokers, and with 398 (44.87%) subjects who had tried to quit smoking previously without success. The mean package year number was 26.6±20.6 (median: 20.0). The mean starting age for smoking was 22.4±9.2. Stratified by gender, 883 men (40.3%) and 4 (0.02%) women reported current or former smoking. Of the 883 male smokers, 701 (79.3%) were current smokers, while of the 4 female smokers 3 still smoked on the examination date. Smokeless tobacco was consumed by 1968 (41.8%) subjects, with 1792 (91.1%) subjects being current users and 176 (8.9%) subjects being former users. Out of the 1968 users of smokeless tobacco, 275 (14.0%) subjects additionally smoked cigarettes/bidis. Gender distribution of the smokeless tobacco users was 1066 men (48.7%) and 902 women (35.8%). A complete Fagerstrom Test for Nicotine Dependence (FTND) could be calculated for 855 smokers (96.4%). The mean of the 855 valid FTND sum scores was 3.87±2.5 points (median: 4). A FTND sum score between 0 and 3 points (low level of addiction) was reported by 424 smokers (49.59%), a score between 4 and 6 points (moderate level of nicotine addiction) was reported by 281 smokers (32.87%), and 150 smokers (17.5%) scored between 7 and 10 FTND points (high level of addiction). Concerning smokeless tobacco no valid sum score was available. For 1957 smokeless tobacco users (99.44%), the mean number of daily consumptions was 4.23±2.2 times (median: 4).

### Alcohol consumption

3628 (77.0%) participants reported to have never drunken alcohol; 287 (6.1%) participants reported on a consumption of alcohol once per month or less often, 264 (5.6%) participants an alcohol consumption of 2–4 times/month, 310 (6.6%) subjects an alcohol consumption of 2–3 times/week; 166 (3.5%) subjects an alcohol consumption of 4 times/week; and 54 (1.1%) subjects declared a daily alcohol consumption. Mean AUDIT sum scores was 1.4±3.8 points (median: 0). For the 1081 individuals reporting to drink alcohol the AUDIT sum score was 6.0±5.8 points (median: 4). An AUDIT sum score of 8 or more (harmful or hazardous drinking) was scored by 283 subjects (6.0% of the study population/26.2% of the individuals consuming alcohol). An AUDIT sum score of ≥13 in women or ≥15 or more in men (alcohol dependence) was achieved by 108 subjects (4.6% of the study population; 51 women (8.9% of women consuming alcohol), 57 men (11.2% of men consuming alcohol).

## Discussion

In our rural Central Indian study, 13.0% of the participants fulfilled the criteria for a major depression and an additional 39.6% of the study population showed mild to moderate depression. Suicide attempt was reported by 199 (4.2%) participants and suicidal thoughts during the last 6 months by 238 (5.1%) participants. Alcohol consumption was reported by 1081 (23.0%) participants; 283 (6.0%) subjects had an AUDIT sum score of ≥8 (harmful or hazardous drinking), and 108 (4.63%) subjects scored an AUDIT sum of ≥13 in women and ≥15 in men indicating alcohol dependence.

The figure of 13.0% for the prevalence of major depression is in agreement with results reported for developing countries by the WHO and the GBD [1], [2]. Previous studies from India estimated the prevalence of depression in Indian community samples [19]–[24]. The most recent and largest population-based study reported by Poongothai and colleagues in 2009 screened more than 24,000 subjects in the city of Chennai (South India) and reported an overall prevalence of depression of 15.1% [19]. Earlier studies which mostly used screening instruments with poorer sensitivity, reported lower frequencies: Nandi and colleagues who performed their studies in the same region in 1972 reported prevalence rates for depression of 5.0% which by 1992 had increased to 7.4% [20]. A review by Math and Chandrashekar in 2006 suggested that the data reported on prevalence rates for psychiatric disorders from epidemiological studies was inconclusive with a high discrepancy in the prevalence rates, ranging from 9.5 to 370/1000 populations in India [23]. They concluded that the prevalence of mental disorders reported in epidemiological surveys could be considered lower estimates rather than accurate reflections of the true prevalence in the population.

The WHO estimated that 170 000 people die of suicide in India out of nearly 900 000 people worldwide, with about another 200 000 subjects in China and 140 000 in high-income countries [25]. In a recent survey on suicide mortality in India, Patel and colleagues found that about 3% of surveyed deaths in individuals aged 15 years or older were due to suicide [26]. A 15-year-old subject in India had a cumulative risk of about 1.3% of dying before the age of 80 years by suicide, with men having a higher risk (1.7%) than had women (1.0%). According to the recent global Burden of Disease Study GBD 2010, suicide was the most common cause of death among Indian women aged between 15 and 49 years, followed by maternal disorders, fire, tuberculosis, and diarrhea [27], [28]. Beside these and other studies on the death rate by suicide in India [29]–[31], none of the major investigations have addressed the prevalence of suicide thoughts and unsuccessful suicide attempts in the Indian population, in particular not in the rural population. A WHO publication based on data collected by India's National Crime Records Bureau, Ministry of Home Affairs showed a rising suicide rate from 1980–2009 [32]. In 1980, there was a total rate of 6.3 suicides per 100.000 people, in 2009 the total rate was 10.5 per 100.000. In this publication the suicide rate for women only showed a slight increase from 1980 to 2009 with a peak in the year 2000, while the rate of suicides among the male population nearly doubled (7.3 in 1980 to 13.0 in 2009).

In the study population, 19% of the individuals reported to be or to have been smokers, with the majority of smokers being men (40.3% of men versus 0.02% of women). Smokeless tobacco was consumed by 1968 (41.8%) subjects. These figures were higher than those reported in the Indian National Family Health Survey-2 from 1998–99 [33]. That survey on 315,598 individuals 15 years or older revealed that 30% of the population (47% men and 14% of women) either smoked or chewed tobacco. It also showed that individuals with no education were 2.7 times more likely to smoke and chew tobacco than those with postgraduate education, and that scheduled tribes had a 23% higher probability to consume tobacco than other social groups [34]. Compared with the figures from a survey focused on 14 low-income and middle-income countries including India, 48.6% of men and 11.3% of women were tobacco users [35]. Again these figures were lower than those for our rural study population. It indicates that our study population had a relatively high prevalence of using tobacco corresponding to the association between lower socio-economic background or lower educational level and tobacco use [34], [36].

Out of our study population, 77.0% of the participants reported to have never drunken alcohol, while an AUDIT sum score of 8 or more (harmful or hazardous drinking) was scored by 6.0% of the study population or 26.2% of the individuals consuming alcohol. In the Indian National Sample Survey as a representative survey of 471,143 people over the age of 10 years in 1995–96 revealed a national prevalence of regular use of smoking tobacco is estimated to be 16.2%, chewing tobacco 14.0%, and alcohol 4.5%. Men were 9.7 times more likely to regularly use alcohol [36]. Respondents belonging to scheduled castes and tribes (recognized disadvantaged groups), people from rural areas and those without formal education were significantly more likely to report regular use of alcohol [37], [38].

Potential limitations of our study should be discussed. First, a major concern in any prevalence study is nonparticipation. The Central India Eye and Medical Study had a reasonable response rate of 80.1%, however, differences between participants and non-participants can lead to a selection artifact. Second, our study included only those who resided in a purely rural region, a region that can be considered to be markedly rural based on responses to the questionnaire regarding socioeconomic background and lifestyle. The study did not include subjects from an urban region, so we can provide no information on any differences between rural and urban regions with respect to the examined parameters. Third, our study as cross-section investigation does not allow firm statements on a longitudinal association, i.e. temporal causation cannot be established with the current cross-sectional design. Fourth, our study only included participants over the age of 30 years and therefore the frequency we report only refer to this age-group. This is important as cohort effects exist for major depression and generally age at onset is declining. Fifth, epidemiological studies using questionnaires such as the CES-D, usually assessed “depressive symptoms” rather than “depression”, so the findings of our study can give information only about depressive symptoms. To make a definitive diagnosis of depression would need additional tests. Sixth, due to the study design, our investigation could not assess relationships between outcome parameters, such as a potential association between smoking and prevalence of depression, or an association between alcohol consumption and depression.

In conclusion, the rural Central Indian population living in a purely agrarian society showed a prevalence of major depression of 13.0% (8.1 of men, 17.3% of women). About 4% of the study population reported about suicide attempt, and 5% indicated to have had suicidal thoughts during the last 6 months. Approximately 20% of the population was present or former smokers. Harmful or hazardous drinking or alcohol dependence was reported by 12% and 5% of the study population, respectively. As in other population and societies, depression was a very common psychiatric disorder. Measures should be taken to address the relatively high prevalence of suicide attempts and thoughts on suicide. Future research focused on the general population, longitudinal (prospective), multi-center, co-morbid studies, assessment of disability, functioning, family burden and quality of life studies involving a clinical service providing approach, is required.
